# TikTok as a platform for hysteroscopy information: An analytical video‐based cross‐sectional study to assess quality, reliability, and accuracy

**DOI:** 10.1002/ijgo.15846

**Published:** 2024-08-09

**Authors:** Salvatore Giovanni Vitale, Stefano Angioni, Stefania Saponara, Gilda Sicilia, Andrea Etrusco, Maurizio Nicola D'Alterio, Luigi Cobellis, Pasquale De Franciscis, Gaetano Riemma

**Affiliations:** ^1^ Division of Gynecology and Obstetrics, Department of Surgical Sciences University of Cagliari Cagliari Italy; ^2^ Department of Health Promotion, Mother and Child Care, Internal Medicine and Medical Specialties (PROMISE) University of Palermo Palermo Italy; ^3^ Unit of Obstetrics and Gynecology “Paolo Giaccone” Hospital Palermo Italy; ^4^ Obstetrics and Gynecology Unit, Department of Woman, Child and General and Specialized Surgery University of Campania “Luigi Vanvitelli” Naples Italy

**Keywords:** healthcare professionals, hysteroscopy, internet, misinformation, patients, quality, reliability, social media, TikTok, video

## Abstract

**Objective:**

To assess the quality, reliability, and level of misinformation in TikTok videos about hysteroscopy.

**Methods:**

A cross‐sectional analysis of TikTok videos retrieved using “hysteroscopy” as search term was performed. Patient education materials assessment tool for audio‐visual content (PEMAT A/V), the modified DISCERN (mDISCERN), global quality scale (GQS), video information and quality index (VIQI) and misinformation assessment were used.

**Results:**

Of three hundred videos captured, 156 were excluded and 144 were included. Most videos were partially accurate or uninformative (43.8% and 34.7%, respectively). Non‐healthcare providers produced more inaccurate or uninformative videos than healthcare workers (51.1% vs 4.0%; *P* < 0.001). Compared to content by professionals, content by patients showed increased distrust towards gynecologists (11.7% vs 0%; *P* = 0.012) and increased incidence of anxiety and concern towards hysteroscopy (25.5% vs 2%; *P* < 0.001). PEMAT A/V scores for understandability and actionability were low at 42.9% (interquartile range [IQR]: 11.1–70) and 0% (IQR: 0–0), respectively. Understandability (*P* < 0.001) and actionability (*P* = 0.001) were higher for professionals' created content relative to patients' videos. Similarly, median mDISCERN score was low (1 [IQR 0–2]), with significantly higher score for healthcare professionals compared to patients (*P* < 0.001). Overall video quality was also low, with median VIQI and GQS score of 7 (IQR 4–11) and 1 (IQR 1–3), respectively, and significantly higher scores for healthcare workers' captions compared to patients' for both (*P* < 0.001 and *P* = 0.001, respectively).

**Conclusion:**

TikTok videos' quality on hysteroscopy seems unsatisfactory and misinformative, with low understandability and actionability scores. Videos recorded by healthcare workers show higher quality and less misinformation than those by patients. Raising the awareness regarding the low quality of medical information on social media is crucial to increase future reliability and trustworthiness.

## Synopsis

Quality of TikTok videos on hysteroscopy appears inadequate, with low scores for actionability and understandability. Effort is required before TikTok can be trustworthy on hysteroscopy.

## 1 INTRODUCTION

Outpatient hysteroscopy is currently regarded as the gold standard for the assessment and treatment of intracavitary uterine pathologies.^1^ Indeed, with technological advancements, hysteroscopic surgery is now considered a minimally invasive procedure with excellent feasibility, low complication rates, and high sensitivity and specificity.^2^ The “see and treat” approach allows for the simultaneous diagnosis and management of various conditions in a single session.^3^ However, when hysteroscopy is performed in an outpatient setting, some patients may still find it to be uncomfortable and difficult to tolerate.^4^

The anxiety that patients sometimes experience prior to undergoing hysteroscopy can exacerbate their perceived discomfort, making the procedure particularly challenging for some individuals.^5^, ^6^ This heightened anxiety may negatively influence patients' overall perception of the procedure and potentially impact their willingness to undergo future outpatient hysteroscopic procedures.^4^

TikTok has recently become one of the most widely visited video‐based social media platforms for sharing and getting health information, with more than one billion monthly active users globally.^7^

In the past, some investigations into the quality and nature of hysteroscopy‐regarding information available on social networks (i.e., YouTube) have been carried out,^8^ yet the characteristics of hysteroscopy‐related content available on TikTok remain largely unexplored.

However, TikTok's platform model, which does not verify content creators' credibility and reliability, potentially allows the circulation of untrustworthy or uninformed content.^9^ This issue is further compounded by the lack of a peer‐review process for the content uploaded on the platform, which makes it possible for registered users to post unchecked media totally at their discretion.^10^, ^11^

Healthcare professionals and government agencies have expressed concerns about the quality and accuracy of readily accessible information on social media, particularly due to the widespread sharing of personal opinions and anecdotal experiences.^12^, ^13^ Such concerns are even more accentuated when it comes to medical procedures—such as hysteroscopy—which are susceptible to misunderstandings and misinformation.^8^

No published studies have examined the hysteroscopy‐related content published on the TikTok platform, despite the procedure's widespread application and TikTok's growing role as a widely accessible resource of easily obtainable healthcare information.

Given the frequent sharing of women's health information on social networks by both healthcare professionals and individuals without gynecologic expertise, the aim of our study was to analyze the characteristics of hysteroscopic content on TikTok and to identify the extent of misinformation and the presence of negative connotations associated with hysteroscopy, shared by a diverse range of users globally.

## 2 MATERIALS AND METHODS

### 2.1 Video selection and data extraction

On January 13, 2024, a search was conducted on TikTok using the keyword “hysteroscopy.” A new account was created for this research to mitigate the influence of algorithmically personalized content. The search output was compiled following the platform's default algorithm, without filtration, ensuring an unbiased selection for preliminary analysis. All videos retrieved under this search term were subjected to a subsequent evaluative process according to predefined inclusion and exclusion criteria.

For inclusion, videos were required to have hysteroscopy as the main focus. Also, videos were required to be presented in English or to be audio‐free. Videos were excluded in case of language other than English, content unrelated to hysteroscopy, and when duplicate material was found.

Data were manually extracted and recorded in a standardized spreadsheet. The collected data included the video's title, duration, description, upload date, hashtags used, geographical location, and engagement metrics such as the number of likes, shares, views, and comments. We also documented the content creators' followers' count, country of origin, total number of video contributions, and the aggregate number of likes received across all their content. The characterization of the main subject or narrator was classified identifying whether they were healthcare professionals (including physicians, nurses, midwives, and medical students) or patients.

The evaluation of video content was conducted by a team composed of one senior gynecologist (SGV) who had performed more than 500 diagnostic and operative hysteroscopic procedures and two residents in Obstetrics and Gynecology (GS and SS). All were staff members of the “Division of Gynecology and Obstetrics, Department of Surgical Sciences” of the University of Cagliari, Italy.

The team members independently reviewed and simultaneously scored the videos to ensure unbiased evaluations. Discrepancies in the assessment were addressed by an additional gynecologist (GR) from the “Department of Woman, Child and General and Specialized Surgery” of the University of Campania “Luigi Vanvitelli”, Naples, Italy. This process subsequently facilitated a unified consensus among all evaluators on the content scores.

### 2.2 Assessment of misinformation, reliability, quality and accuracy

Each video was evaluated through the following standardized tools. We evaluated the interpretability and practicability of the educational content using the patient education materials assessment tool for audiovisual content (PEMAT A/V). This tool consists of 17 items evaluating the content's understandability (items 1–13) and actionability (items 14–17). Higher percentages reflect content deemed more understandable and/or actionable.^14^

To evaluate the reliability of the video content, we utilized the modified DISCERN (mDISCERN) scale. Initially developed by Charnock et al., it consists of five questions.^15^ Each question answered affirmatively contributes a single point to a total possible score of five. The five questions on the mDISCERN scale are as follows: (1) Are the aims clear and achieved? (2) Are reliable sources of information used? (i.e., publication cited, the speaker is a specialist). (3) Is the information presented balanced and unbiased? (4) Are supplementary sources of information indicated for the benefit of the patient? (5) Are areas of uncertainty mentioned? A higher mDISCERN score suggests increased reliability, with scores of three or higher considered to reflect a high degree of reliability.^16^

The video content's quality was evaluated using the global quality scale (GQS). The original purpose of this assessment instrument was to measure the utility, structure, and accessibility of web‐based resources. According to the GQS, videos are rated on a scale from one to five.^17^ A higher GQS score corresponds to content of superior quality and informative value. Videos that received a GQS rating of three or above were considered to provide high‐quality health information.^18^

Additionally, the overall educational quality and substance of the videos were examined using the video information and quality index (VIQI). The VIQI is a 5‐point rating system that consists of four rating criteria: video quality (VIQI 3), information flow (VIQI 1), clarity (VIQI 2), and video consistency (alignment of the video's title and content) (VIQI 4).^19^

Each video underwent a rigorous fact‐checking process to pinpoint and address any erroneous information presented. This verification was conducted utilizing accepted guidelines for hysteroscopy and data from the existing literature. The misinformation level within the videos was categorized as follows: content that included more than 50% false information was classified as “inaccurate or misinformative”; videos containing less than 50% but more than 25% false information were classified as “partially accurate”; videos containing less than 25% misinformation were labeled as “accurate and evidence‐based.”

### 2.3 Statistical analysis

The data's normality was examined using the Shapiro–Wilk test. The median and interquartile range (IQR) statistical measures were used to characterize non‐normally distributed data, whereas frequency and percentage were used to convey counting data.

When comparing variables across groups, the Kruskal Wallis test was utilized to assess differences and Dunn's test was employed when comparing variables that were not normally distributed in two ways between groups. Statistical significance was defined as a *P* value less than 0.050. Our method of evaluating the link between non‐normal variables was Spearman correlation analysis. All statistical analyses were performed using STATA 14.1 (StataCorp LLC, College Station, TX, USA).

### 2.4 Ethical approval

Institutional Review Board approval was not required since the study did not include human participants or interventions; all included information is publicly available on TikTok.

## 3 RESULTS

### 3.1 Videographic characteristics

**FIGURE 1:**
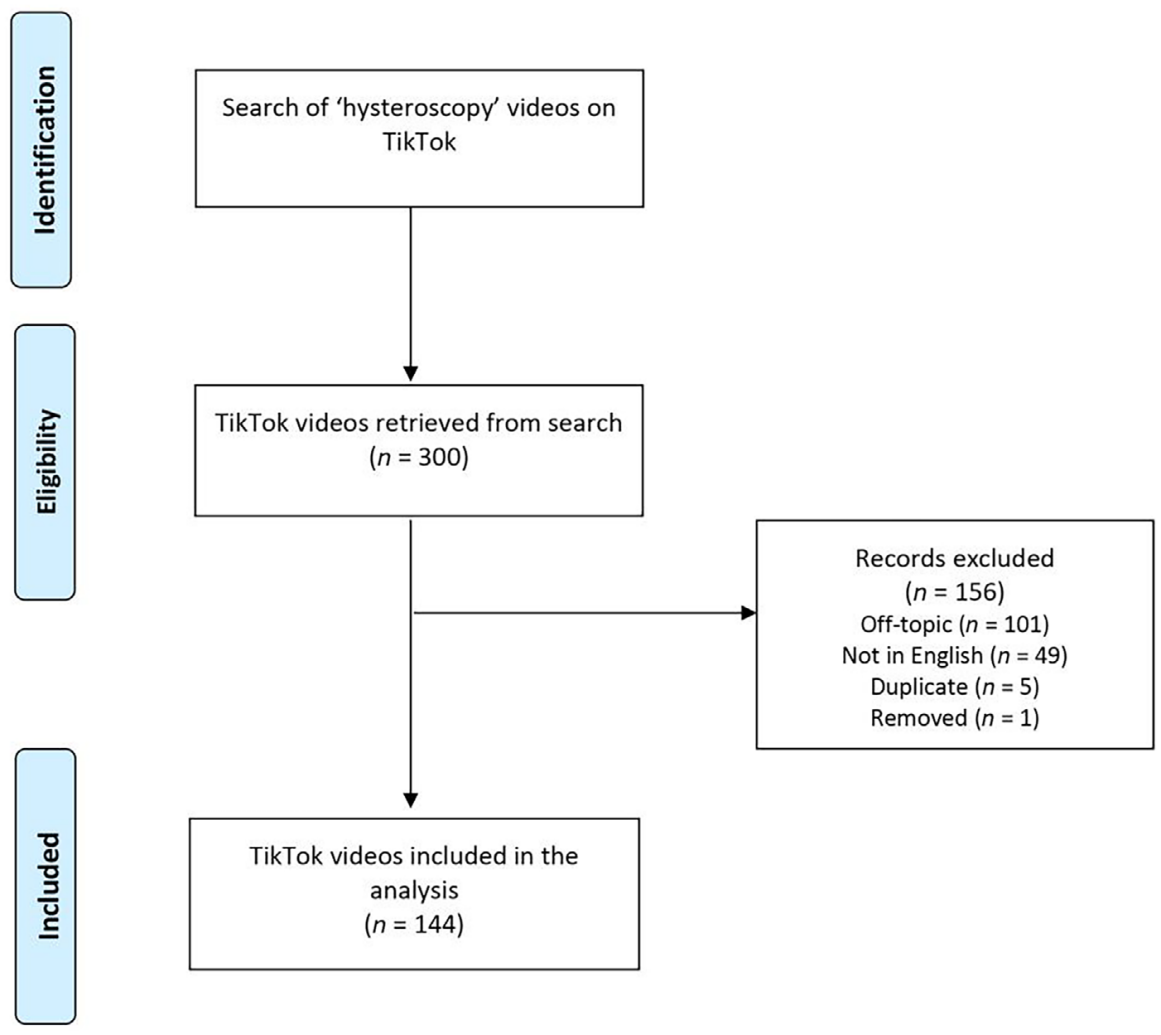
Flow chart depicting the selection process of TikTok videos.

Of the 300 videos reviewed, 144 were deemed suitable for analysis (Table 1). A total of 156 videos were excluded: 101 were off‐topic, 49 were recorded in a language other than English, one was removed from the platform, and five were identified as duplicates (Figure 1).

**TABLE 1:** Videographic characteristics of TikTok videos regarding hysteroscopy included in the analysis.

Video characteristics			Healthcare workers (50)	Patients (94)	*P* value
Length (s)	Median	51	51	51	0.076
	(IQR)	(22.5–91.5)	(30–96)	(22–91)	
	Range	1–528	1–179	1–528	
Views	Median	6494	12 700	5726	**0.002**
	(IQR)	(2562.5–19 250)	(3377–42 600)	(2290–171 400)	
	Range	484–907 500	484–300 000	497–907 500	
Comments	Median	12.5	12	14	0.584
	(IQR)	(3–34)	(4–36)	(3–32)	
	Range	0–1711	0–200	0–1711	
Likes	Median	98.5	403	246	0.132
	(IQR)	(44–277.5)	(86–3886)	(44–246)	
	Range	5–55 900	5–10 400	5–55 900	
Shares	Median	3	6	4	**0.001**
	(IQR)	(2–9)	(3–31)	(2–17)	
	Range	0–592	1–369	0–592	
Followers	Median	5285.5	14 300	3484	**0.001**
	(IQR)	(1413.5–35 650)	(1503–71 900)	(1159–19 300)	
	Range	13–1 200 000	13–412 900	53–1 200 000	
Author likes	Median	103 500	164 100	85 600	0.134
	(IQR)	(10120–549 750)	(14200–571 500)	(7776–427 800)	
	Range	116–37 200 000	116–1 360 000	161–37 200 000	
Location	N.A.	97 (67.4)	31 (62)	66 (70.2)	**0.001**
	S.A.	0 (0)	0 (0)	0 (0)	
	Europe	24 (16.7)	7 (14)	17 (18.1)	
	Africa	8 (5.6)	4 (8)	4 (4.3)	
	Asia	12 (8.3)	9 (18)	3 (3.2)	
	Oceania	3 (2.1)	0 (0)	3 (3.2)	

Abbreviations: IQR, interquartile range; N.A, North America; S.A, South America.

Bold values indicates *p*‐value less than 0.050

The videos had a median length of 51 s (IQR: 22.5–91.5; range: 1–528). The median number of views was 6494 (IQR: 2562.5–19 250; range: 484–907 500). Median number of comments, likes, shares, followers and total likes are reported in Table 1.

Fifty videos (25.5%) were produced by healthcare providers, while 94 (74.5%) were recorded or narrated by patients. All the videos were targeted to a patient‐based audience. The content generated by healthcare professionals gained significantly more views, shares, and followers than that produced by patients (Table 1).

Table 2 describes the general characteristics of the videos analyzed. Regarding the video category, healthcare professionals provided more medical education content, while patients tended to share personal experiences (Table 2; *P* < 0.001). Similarly, the video's tone in the content produced by healthcare professionals was generally more neutral than that used by patients (Table 2; *P* < 0.001).

**TABLE 2:** General characteristics and misinformation assessment method of included videos.

Overall			Healthcare workers (50)	Patients (94)	*P* value
Setting	*N* (%)				0.068
Operating room (with anesthesia)		43 (29.9)	10 (20)	33 (35.1)	
Office/outpatient (without anesthesia)		19 (13.2)	5 (10)	14 (14.9)	
N/A		82 (56.9)	35 (70)	47 (50)	
Video category	*N* (%)				**<0.001**
Entertainment		4 (2.8)	1 (2)	3 (3.2)	
Medical education		62 (43.1)	49 (98)	13 (13.8)	
Patient's complaint		11 (7.6)	0 (0)	11 (11.7)	
Storytelling		67 (46.5)	0 (0)	67 (71.3)	
Tone of the video	*N* (%)				**<0.001**
Positive		57 (39.6)	17 (34)	40 (42.6)	
Negative		21 (14.6)	0	21 (22.3)	
Neutral		66 (45.8)	33 (66)	33 (35.1)	
Purpose	*N* (%)				0.145
To educate or inform		116 (80.6)	44 (88)	72 (76.6)	
To encourage the patient in self‐defense		5 (3.5)	0	5 (5.3)	
Persuade to avoid hysteroscopy.		4 (2.8)	0	4 (4.3)	
Reassure or encourage the use of hysteroscopy		19 (13.2)	6 (12)	13 (13.8)	
Misinformation assessment	*N* (%)				**<0.001**
Accurate and evidence‐based		31 (21.5)	28 (56)	3 (3.2)	
				4	
Partially accurate		63 (43.8)	20 (40)	3 (45.7)	
Inaccurate or uninformative		50 (34.7)	2 (4)	48 (51.1)	
Does it describe alternatives to hysteroscopy?	*N* (%)				0.093
No		138 (95.8)	46 (92)	92 (97.9)	
Yes		6 (4.2)	4 (8)	2 (2.1)	
Does it suggest that hysteroscopy is painful?	*N* (%)				**0.002**
No		119 (82.6)	48 (96)	71 (75.5)	
Yes		25 (17.4)	2 (4)	23 (24.5)	
Does it encourage patients to avoid hysteroscopy?	*N* (%)				0.068
No		138 (95.8)	50 (100)	88 (93.6)	
Yes		6 (4.2)	0 (0)	6 (6.4)	
Does it encourage patients to choose hysteroscopy?	*N* (%)				**0.009**
No		108 (75.0)	31 (62)	77 (81.9)	
Yes		36 (25.0)	19 (38)	17 (18.1)	
Does it encourage patients to distrust their gynecologist?	*N* (%)				**0.012**
No		133 (92.4)	50 (100)	83 (88.3)	
Yes		11 (7.6)	0 (0)	11 (11.7)	
Does it increase anxiety/concern about hysteroscopy?	*N* (%)				**<0.001**
No		119 (82.6)	49 (98)	70 (74.5)	
Yes		25 (17.4)	1 (2)	24 (25.5)	

Bold values indicates *p*‐value less than 0.050

Patients referred to hysteroscopy as a painful examination significantly more than healthcare workers (23 [24.5%] vs 2 [4.0%]; *P* = 0.002) and were less likely to recommend hysteroscopy when necessary (17 [18.1%] vs 19 [38%]; *P* = 0.009). Nonetheless, although with low percentages, patients' content showed increased distrust towards the gynecologist compared to healthcare professionals' videos (11 [11.7%] vs 0 [0%]; *P* = 0.012). Moreover, videos carried out by patients had a significant incidence of anxiety‐related content compared to those created by healthcare professionals (24 [25.5%] vs 1 [2%]; *P* < 0.001) (Table 2).

### 3.2 Misinformation assessment

Concerning the misinformation about hysteroscopic content on TikTok, most of the content was considered partially accurate or uninformative (63 [43.8%] and 50 [34.7%], respectively). When subcategorized according to the author, there was a significant difference between healthcare professionals and patients, with more inaccurate or uninformative videos recorded by non‐healthcare providers (48 [51.1%] vs 2 [4.0%]; *P* < 0.001) (Table 2).

### 3.3 Quality assessment

The overall median PEMAT A/V understandability and actionability scores were 42.9% (IQR: 11.1–70.0) and 0% (IQR: 0.0–0.0). According to the creator's role, the median understandability score was statistically significantly higher for videos made by healthcare workers, relative to patients (60.0% [IQR: 33.3–85.7] vs 33.3% [IQR: 11.1–60.0], *P* < 0.001). Moreover, although with a median of 0 for both groups, due to different IQRs, videos recorded by healthcare workers had a higher actionability score of the PEMAT A/V tool compared to patients' videos (0% [IQR: 0.0–33.3] vs 0% [IQR: 0.0–7.0], *P* = 0.005) (Table 3).

**TABLE 3:** Quality assessment according to PEMAT A/V, mDISCERN, VIQI and GQS.

Video characteristics			Healthcare workers (50)	Patients (94)	*P* value
PEMAT A/V					
Understandability %	Median	42.9	60	33.3	**<0.001**
	(IQR)	(11.1–70)	(33.3–85.7)	(11.1–60)	
	Range	0–100	0–100	0–100	
Actionability %	Median	0	0	0	**0.005**
	(IQR)	(0–0)	(0–33.3)	(0–0)	
	Range	0–100	0–100	0–66.7	
mDISCERN	Median	1	2	1	**<0.001**
	(IQR)	(0–2)	(1–3)	(0–2)	
	Range	0–4	0–4	0–3	
VIQI					
Flow (VIQI1)	Median	2	2	1	**<0.001**
	(IQR)	(1–2)	(2–3)	(1–2)	
	Range	1–5	1–5	1–4	
Information accuracy (VIQI 2)	Median	2	3	1	**<0.001**
	(IQR)	(1–3)	(2–4)	(1–2)	
	Range	1–5	1–5	1–4	
Quality (VIQI 3)	Median	2	2	1	**<0.001**
	(IQR)	(1–2)	(1–2)	(1–2)	
	Range	1–5	1–5	1–4	
Precision (VIQI 4)	Median	2	3	1	**<0.001**
	(IQR)	(1–4)	(2–4)	(1–3)	
	Range	1–5	1–5	1–5	
VIQI score (total)	Median	7	11	5	**<0.001**
	(IQR)	(4–11)	(6–14)	(4–9)	
	Range	4–20	4–20	4–16	
GQS	Median	1	2	1	**0.001**
	(IQR)	(1–3)	(1–3)	(1–2)	
	Range	1–4	1–5	1–4	

Abbreviations: GQS, global quality scale; mDISCERN, modified DISCERN; PEMAT A/V, patient education materials assessment tool for audio‐visual content; VIQI, video information and quality index.

Bold values indicates *p*‐value less than 0.050

The median mDISCERN score was 1 (IQR: 0–2), with a significantly higher score for physicians' created content relative to patients' videos (2 [IQR: 1–3]; vs 1 [IQR: 0–2]; *P* < 0.001) (Table 3).

The overall score for VIQI showed low‐level content, with a median score of 7 (IQR: 4–11). Videos produced by healthcare workers scored significantly higher score than those produced by patients (11 [IQR: 6–14] vs 5 [IQR: 4–9]; *P* < 0.001). This trend was consistent across all VIQI subcategories, including flow (VIQI1), information accuracy (VIQI 2), quality (VIQI 3), and precision (VIQI 4), with videos from non‐physicians scoring significantly lower. (all *P* < 0.001; Table 3).

Similarly, low scores were reported for the GQS in the overall healthcare workers and patients' assessment, with increased scores for videos created by healthcare professionals‐made videos (median 2 [IQR: 1–3] vs 1 [IQR: 1–2]; *P* = 0.001) (Table 3).

### 3.4 Variable correlations

We recorded a positive statistically significant correlation between PEMAT A/V understandability and number of shares (*r* = 0.15; *P* = 0.010), author's followers (*r* = 0.13; *P* = 0.021), and author's likes (*r* = 0.12; *P* = 0.040). Similarly, the PEMAT A/V actionability was significantly correlated with the number of comments (*r* = 0.12; *P* = 0.040); the mDISCERN was significantly correlated with the number of likes (*r* = 0.14; *P* = 0.027), shares (*r* = 0.32; *P* < 0.001), views (*r* = 0.26; *P* < 0.001), author's followers (*r* = 0.26; *P* < 0.001), and author's likes (*r* = 0.17; *P* < 0.001). The VIQI tool had a significant correlation with the video length (*r* = 0.12; *P* = 0.030), number of shares (*r* = 0.25; *P* < 0.001), views (*r* = 0.19; *P* = 0.010), and author's followers (*r* = 0.16; *P* = 0.010); meanwhile, the GQS had a significant correlation with video length (*r* = 0.13; *P* = 0.021), number of likes (*r* = 0.13; *P* = 0.030), shares (*r* = 0.25; *P* < 0.001), views (*r* = 0.201; *P* = 0.001), author's followers (*r* = 0.21; *P* < 0.001) and author's likes (*r* = 0.16; *P* = 0.010). On the other hand, no associations were found between the quality video assessment tools and any of the other videographic attributes (all *P* > 0.050).

## 4 DISCUSSION

This cross‐sectional analysis revealed that the reliability and quality of TikTok videos on hysteroscopy are generally low. Additionally, many videos contain inaccurate or partially accurate information, posing a significant risk of spreading misinformation. Videos produced by healthcare professionals tended to be clearer, of higher quality, and contained fewer inaccuracies and misinformation compared to those created by patients.

During the analysis, we identified several noteworthy observations. Among the 144 videos included for the analysis, a median of 6494 views were recorded, which is inherently low due to the limited public interest in hysteroscopy compared other gynecologic topics (e.g., in vitro fertilization [IVF]). This was juxtaposed with a median of 12.5 comments, 98.5 likes and three shares, highlighting low user engagement with the current hysteroscopy content on TikTok. This observation may result from non “catchy” video content, insufficient high‐quality videos in terms of videographic characteristics or viewer engagement, and subpar production values.

According to the PEMAT A/V score, the overall median understandability was 42.9% and the overall median actionability was 0%. The understandability score measures the ease with which viewers comprehend the information displayed in the videos, while the actionability score reflects the potential for viewers to apply the information practically. According to Shoemaker et al.,^20^ a PEMAT score <70% is considered poorly understandable or actionable. Consequently, we recorded poorly understandable and actionable content based on our results. Specifically, the median actionability score of 0% for healthcare workers and patients should be related to the fact that most of the videos were not recorded to demonstrate a step‐by‐step approach to performing hysteroscopy but to increase patient awareness about the procedure and its associated concerns or complications. In fact, several videos were patient's experience of hysteroscopic procedures, and while such content is indeed useful for clinicians to understand the woman's perspective of the examination, in terms of actionability, information accuracy, video quality and precision of the information, it contributed to lowering the scores calculated using the above‐mentioned tools. A higher number of videos made by healthcare professionals would have probably increased the level of the overall quality assessment.

In contrast, videos recorded by healthcare professionals achieved a median understandability score of 60%, while videos recorded by patients had a median score of 33.3%. This suggests that while neither group's videos were highly actionable, those created by healthcare professionals were closer to being understandable. Videos created by patients, on the other hand, were lacking in both clarity and practical usefulness.

No prior researchers investigated the content quality of hysteroscopy‐related information shared on TikTok. Our results align with existing literature assessing the integrity and engagement of medical content on TikTok across various specialties. Irfan et al.^21^ discovered that physician‐created content on acne garnered more user engagement metrics such as views, likes, comments and shares than non‐physician‐created content. Videos with the greatest DISCERN score shared personal experiences despite an overarching trend of poor DISCERN scores.^21^ These results suggest that dermatologists are perceived as reliable sources of TikTok for information on acne. However, Irfan et al.^21^ underscored the crucial role of all health experts in advancing effective health communication in the digital realm by offering trustworthy, evidence‐based information on these platforms.^21^ In their examination of ophthalmologic content on TikTok, Sampige et al.^14^ found that a lot of the popular content on the platform was made by non‐specialists and had inaccurate information. Their findings confirmed that, in order to stop the spread of misleading information, ophthalmologists need to create more factual, useful, and interesting teaching materials.^14^

Conversely, Kanner et al.^22^ analyzed the actionability, understandability, and quality of overactive bladder‐related content, finding that TikTok videos scored low on PEMAT understandability.^22^

Collectively, these studies point to the importance of improving content quality on TikTok and to the need for expert involvement in content creation to ensure accuracy and usefulness. There was a discernible difference in the videos' understandability and actionability scores, contingent on the topic and the expertise of the content creator. It is advisable for TikTok and other social networks, to raise the awareness regarding the quality of the information retrievable in the uploaded content. To improve such issue, it would be advisable to brand the medical content created by official and ascertained professionals as reliable and trustworthy by the platform itself.

### 4.1 Strengths and limitations

The present study stems from the idea of assessing the quality of TikTok videos regarding hysteroscopic examinations, specifically in the context of outpatient care, and to determine whether patients could dispel their myths and learn notions of solid scientific value from these short videos. As far as we know, no research has been conducted on this goal. We closed this gap and discovered several intriguing elements. This attribute is one of our study's strengths since hysteroscopy is a key procedure for several gynecologic issues and social medias are often the fastest sources of information for women undergoing the examination.^23^, ^24^, ^25^

There are, however, a few limitations to take into consideration. First, the video analysis group was composed only of gynecologic‐related professionals; a representation of patients in the team would have increased the heterogeneity of the evaluation and decreased the possibility of selection and response biases. The video selection process, confined by the TikTok algorithm's keyword sensitivity, may have represented an element of bias, potentially affecting the breadth and applicability of our findings. The specificity of search terms, algorithms, and selection criteria might have skewed the representation of different healthcare settings, professional backgrounds and content categories. For example, the search was limited to the keyword “hysteroscopy,” which meant that relevant videos tagged with different keywords were not found.

Moreover, it should be remarked that the TikTok algorithm still needs improvement regarding the selection of search results based on queried keywords. For this purpose, of the 300 videos captured by searching the keyword “hysteroscopy” 101 were deemed off‐topic but still included in the search results. Of these, 56 (55.4%) were related to non‐hysteroscopic gynecologic topics, four (4.0%) to obstetrical content and four (4.0%) to non‐gynecologic endoscopic procedures. The remainder consisted of 39 (38.6%) videos that were completely off‐topic, albeit dealing with medical topics.

## 5 CONCLUSIONS

To date, TikTok videos on hysteroscopy are not a reliable source of correct information in terms of understandability and applicability. Moreover, due to the high level of misinformation retrievable, the reliability, accuracy, and quality of the content is relatively low. Videos created by healthcare professionals generally demonstrated enhanced reliability and understandability and were of higher quality than those produced by patients. As high levels of misinformation were reported and social media are frequently visited sources of information by women undergoing hysteroscopy or other gynecologic procedures, TikTok creators (both physicians and non‐physicians) and the platform itself are encouraged to provide more trustworthy and accurate content to their followers to mitigate the propagation of false myths and misconceptions regarding hysteroscopy and to guarantee valuable health education standards on this widely utilized social media.

## AUTHOR CONTRIBUTIONS

Salvatore Giovanni Vitale and Gaetano Riemma: Designed the study and wrote the manuscript. Gilda Sicilia, Andrea Etrusco and Stefania Saponara: Performed data curation, data analysis and revised the manuscript. Gaetano Riemma and Maurizio Nicola D'Alterio: Performed statistical analyses. Luigi Cobellis, Pasquale De Franciscis, Stefano Angioni, Stefania Saponara, Andrea Etrusco and Gilda Sicilia: Critically revised the manuscript. Gaetano Riemma and Salvatore Giovanni Vitale: Interpreted data and drafted the manuscript. All authors read and approved the final manuscript.

## FUNDING INFORMATION

This study did not receive any grant or funding.

## CONFLICT OF INTEREST STATEMENT

The authors declare that they have no conflicts of interest to disclose regarding this publication.

## Data Availability

Data sharing is not applicable to this article as no new data were created or analyzed in this study.
